# Health System–Level Implementation of Digital Health Support for People Living With HIV and Substance Use Disorders: Protocol for a Cluster-Randomized, Stepped-Wedge Clinical Trial

**DOI:** 10.2196/69842

**Published:** 2025-08-29

**Authors:** Ryan P Westergaard, Hailey K Ballard, Rachel E Gicquelais, Cynthia Firnhaber, Rebecca Miller, Melanie Wolman, Cameron Liebert, Emily Steeley, Marlon P Mundt, Linda S Park, Andrew Quanbeck

**Affiliations:** 1 Department of Population Health Sciences School of Medicine and Public Health University of Wisconsin-Madison Madison, WI United States; 2 Department of Medicine School of Medicine and Public Health University of Wisconsin-Madison Madison, WI United States; 3 Vivent Health Denver, CO United States; 4 Anschutz School of Medicine University of Colorado Denver, CO United States; 5 Department of Family Medicine and Community Health School of Medicine and Public Health University of Wisconsin-Madison Madison, WI United States

**Keywords:** HIV, antiretroviral agents, substance-related disorders, medication adherence, digital health, case management, mobile phone

## Abstract

**Background:**

People living with HIV who are affected by substance use disorders and other social vulnerabilities are less likely to achieve and sustain viral suppression. Supporting these patients with mobile health systems can provide additional social and behavioral support that may improve HIV outcomes in these populations.

**Objective:**

This study aims to implement and evaluate an evidence-based digital health system (antiretroviral therapy care coordination; ART-C) to improve HIV viral suppression and reduce missed clinic visits within a multisite HIV care program.

**Methods:**

ART-C will be implemented in 8 HIV medical home clinics operated by Vivent Health in Colorado, Missouri, Texas, and Wisconsin. All patients receiving HIV care across the system will be invited to use a customized version of the Connections smartphone app to join a virtual community of peers living with HIV. A subset of patients with substance use disorder will be recruited to use enhanced features of the app, allowing sharing of information about substance use and other social determinants of health with their care team. Effectiveness and implementation outcomes will be evaluated using a stepped-wedge clinical trial design. Effectiveness will be evaluated by comparing missed visits and viral nonsuppression rates before and after the intervention. Implementation will be evaluated using a mixed methods approach based on the reach, effectiveness, adoption, implementation, and maintenance framework.

**Results:**

Funding was received April 2022 with data collection beginning December 6, 2023. Data collection is anticipated to end in 2027 with data analysis and results expected late 2027. As of submission of the manuscript, 86 participants were recruited. Of the 6710 patients with health record data available 2 years before the trial began, 1723 (25.68%) had a substance use disorder, 2825 (42.1%) missed at least 2 care visits, and 1354 (20.18%) had at least 1 detectable HIV RNA test (viral load ≥200 copies/mL), leaving 3816 (56.87%) eligible individuals. Analysis of preimplementation data demonstrated that patients had a mean of 4.1 (SD 2.1) to 7.8 (SD 5.5) care visits across all 8 Vivent Health sites, with 45% to 70% of patients missing at least 1 visit. Patients underwent a mean of 2.2 to 4 HIV RNA tests, and 14% to 37% of patients across clinics had at least 1 nonsuppressed HIV RNA result. Primary analyses are expected to be completed by 2028.

**Conclusions:**

This study will evaluate the effectiveness of and implementation strategies for a mobile health–based intervention to support patients with HIV, substance use disorder, and related challenges in engaging in care and achieving viral suppression.

**International Registered Report Identifier (IRRID):**

DERR1-10.2196/69842

## Introduction

### Background

Clinical implementation and global scale-up of antiretroviral therapy (ART) for HIV have transformed one of the deadliest pandemics in human history into a chronic disease management challenge. Before 2003, HIV and AIDS were a leading cause of death among young adults worldwide. In recent decades, however, effective treatment and systems of care have demonstrated such an impact that the World Health Organization has declared HIV elimination to be a realistic global health goal [1].

Elimination of HIV transmission through effective treatment (also referred to as “treatment as prevention”) can only be achieved through universal access to treatment and sustained viral suppression (defined as plasma HIV RNA levels that are lesser than the detection threshold of 200 copies/mL) for nearly all persons living with HIV [2]. Unfortunately, in the United States, only 62.7% of persons diagnosed with HIV infection had viral suppression in 2017 [3], a level that falls substantially short of the 95% goal set by the US Department of Health and Human Services for ending the epidemic [4].

Patients and communities impacted by substance use disorders (SUDs) and related social and behavioral vulnerabilities are significantly less likely to experience sustained HIV suppression [5-8]. In a cohort of people who inject drugs followed for a median of 8.7 years, only 30% were retained in care every year they were in the study [9]. Of those who reported receiving ART during the study, 86% achieved viral suppression during at least 1 study visit; however, only 12% were found to have achieved viral suppression at every study visit after ART initiation, meaning nearly 9 in 10 patients experienced virologic failure during their treatment [9].

Previous research has demonstrated that the determinants of suboptimal HIV treatment outcomes are heterogeneous and complex [10], suggesting that ending the HIV epidemic will require the implementation of multifaceted strategies to support vulnerable patients throughout their life course. Fortunately, systems of HIV care that address social determinants of health can help address these complex needs. Data from the Health Resources and Services Administration (HRSA) Ryan White HIV and AIDS Program (RWHAP) [11] showed that 85% of individuals who had at least 1 medical visit to a clinic funded by RWHAP during 2017 had achieved viral suppression [12]*.* A study on service delivery in RWHAP-funded facilities found that they were more likely to provide case management, mental health, substance use, and other support services compared to non–RWHAP-funded clinics, and patients attending these facilities were more likely to receive these services [13].

### The Need for Just in Time Strategies for Monitoring and Responding to Social Determinants of Health in HIV Care Settings

While clinical factors, such as antiretroviral adherence [14] and retention in care [15] are well understood to be proximal determinants of viral suppression, nonclinical factors, such as poverty [16], lack of health insurance [17], homelessness [18], food insecurity [19], and criminal justice involvement, also influence HIV care outcomes. In 1 community-based cohort study of predominantly African American people with HIV who inject drugs, participants who achieved viral suppression after initiating ART had a 1-in-4 risk of experiencing viral rebound (ie, virologic failure) during any 6-month interval [20]. In that study, loss of viral suppression was more common if participants reported recent incarceration, daily injection drug use, or homelessness, demonstrating how time-varying social and behavioral factors can signal vulnerability to treatment interruptions Other patient-level factors that have been consistently correlated with treatment interruptions include depression and anxiety [21], low self-efficacy, internalized HIV-related stigma [22], and inadequate social support [23].

A major challenge to addressing HIV-related health disparities is the tendency for patients to miss appointments during periods of greatest vulnerability. Research using electronic health record (EHR) data shows that treatment failure is predictable based on individual-level characteristics documented in the EHR [24,25]. However, the social, behavioral, and structural factors that increase the risk of treatment disruption and require the timely provision of support to ensure care retention are not typically captured in EHRs. Therefore, health care providers do not generally have timely access to data that would help them provide support when it is most needed. As a result, most disruptions in ART are detected only after a lapse in care has already occurred. The fact that previous treatment disengagement and virologic nonsuppression are 2 strong predictors of future care disengagement [24,25] further underscores the need to interrupt the cycle of treatment disruption and find new ways to support retention.

### Digital Health Approaches to Addressing Disparities in HIV Care

Real-time monitoring of patients with digital health systems can provide a platform to provide timely support and linkage to needed services. A suite of demonstration projects supported by HRSA’s Special Projects of National Significance explored the beneficial roles of social media and mobile health (mHealth) interventions, finding promise in three domains: (1) managing HIV care with subthemes of medical information accessibility, reminders, and self-efficacy; (2) fostering feelings of support and personal connectedness; and (3) helping alleviate negative feelings about status and mitigating HIV-related stigma [26]. Technology can empower patients with direct delivery of individualized motivation, education, and support [27]. Wireless technologies remove the barriers of time and distance between patients and health care providers; this is especially important for patients with SUD and other populations that are hard to reach and difficult to keep engaged in care. Digital health technology offers great potential for collecting higher-quality data related to care delivery and health outcomes, and facilitating more effective collaboration among HIV care providers.

In this paper, we describe a hybrid implementation effectiveness trial of a clinic-based intervention that seeks to leverage the numerous potential benefits of digital health strategies when deployed in the context of comprehensive, patient-centered HIV care. The approach described in the Methods section was informed by a pilot study funded by the National Institute on Drug Abuse, in which 200 people living with HIV were invited to use a digital health app, the Addiction–Comprehensive Health Enhancement Support System (CHESS), for a period of 2 years [28,29]. Lessons learned from the pilot study influenced the design of key intervention elements in this study, including the use of peer mentors to boost patient engagement with the digital health system and dedicated clinic case managers to act as liaisons between app engagement and the broader HIV care team.

## Methods

### Overview

The goal of this project is to implement and evaluate an evidence-based digital health system to improve HIV viral suppression and reduce missed clinic visits within a multisite, comprehensive HIV care program in the United States. Using a stepped-wedge, cluster randomized trial design, we will test the effectiveness of a care coordination intervention named ART-C (antiretroviral therapy care coordination). The intervention is a multicomponent strategy that combines medical case management; peer support; and the Connections app, which is a smartphone-based platform designed to support engagement in care for people living with SUD. After conducting formative research and a community-engaged protocol development process, the trial was approved by the institutional review board (IRB) in October 2023. Protocol version 4 was approved on November 14, 2024. In this paper, we discuss the intervention, study aims and methods, and the population eligible for recruitment into the clinic-supported arm of the study.

The Connections App (CHESS Health) is based on the CHESS, a mHealth system developed at the University of Wisconsin (UW). CHESS is a suite of secure, internet-based services delivered via smartphone that promotes positive behavior change and provides social support to people engaged in care for HIV, addiction, and other complex conditions. A randomized controlled study conducted in 2014 found that Addiction–CHESS, a derivation of CHESS designed for alcohol use disorder treatment, had a significant effect on treatment retention and reduced risky drinking behaviors [30].

When implemented in a multisite HIV care program operating under a patient-centered medical home model, we hypothesize that ART-C will improve care by supporting three needs: (1) facilitating real-time, community-based capture of data reflecting social and behavioral determinants known to precede lapses in HIV care (eg, housing and food insecurity, unhealthy alcohol or drug use, or poor medication adherence); (2) improving engagement in care by increasing social connectedness among patients and between patients and peer mentors; and (3) supporting retention in addiction treatment and mental health care that promote sustained engagement in HIV care.

### Study Aims

As a hybrid type 2 trial [31], the study has primary aims related to both effectiveness and implementation. The effectiveness-related aim of the study is to understand the impact of ART-C on the occurrence of virologic failure and missed clinic appointments among patients receiving HIV care. The implementation-related aim is to evaluate the deployment of ART-C within the HIV medical home model using the reach, effectiveness, adoption, implementation, and maintenance (RE-AIM) framework. We will analyze patient-, health care provider-, and clinic-level factors that influence the reach, effectiveness, implementation, adoption, and maintenance of ART-C within HIV care practices.

The hybrid trial design will simultaneously test the effectiveness of the ART-C intervention and the effect of a bundle of evidence-based implementation strategies. Our bundled implementation strategy is organized by the exploration, preparation, implementation, and sustainment framework [32]. The discrete implementation strategies that make up the bundle come from the Expert Recommendations for Implementing Change taxonomy [33].

### Study Design

The stepped-wedge design is a cluster-randomized, 1-way crossover study in which all units are initially assigned to the control condition, then switched to the intervention at randomly assigned time points until all units have crossed over by the last time interval. Stepped-wedge trials enable all sites to receive enhanced services while avoiding biases associated with uncontrolled pre- and poststudy designs. A site will be considered preintervention when it is in the control condition (before ART-C implementation), and postintervention after crossover. Primary outcomes will be passively collected using data from the health system’s EHR.

### Study Setting and Patient Population

ART-C will be implemented within 8 HIV medical home clinics operated by Vivent Health in Colorado, Missouri, Texas, and Wisconsin. The HIV medical home model provides HIV-oriented comprehensive care to people living with HIV and encompasses primary health care, dental care, mental and behavioral health services, case management, housing, prevention or harm reduction services, and food bank services.

To describe the baseline, preintervention occurrence of study outcomes, we defined a cross-sectional sample of active patients who would be eligible to receive ART-C as of December 2023. The baseline sample excluded patients who never attended a medical visit, died before the time of study approval, or did not speak English.

Demographic and clinical characteristics of the study sample were captured from the Vivent Health EHR (Epic Systems Corporation). The prevalence of SUD was determined based on visit diagnoses and problem list entries in the EHR, using the *International Classification of Diseases, 10th Revision,* codes (F10-16, F19, O35.4, O35.5, O99.31, and O99.32). Patients were also classified as having SUD if they were ever prescribed a medication to treat alcohol use disorder (acamprosate, disulfiram, topiramate, or naltrexone) or opioid use disorder (methadone, buprenorphine, or naltrexone). For medications that are used for the treatment of SUD and other conditions, patients were only considered to have SUD if the prescription order was paired with an appropriate diagnosis code for alcohol use disorder or opioid use disorder.

### Study Outcomes

We defined 2 study outcomes using EHR data, which will be examined at the clinic level and continually assessed during pre- and postimplementation periods. The first outcome, missed clinic visits, is defined as the proportion of appointments scheduled with an HIV care provider that a patient failed to attend and did not call in advance to cancel or reschedule. The second outcome, viral nonsuppression, is defined as the proportion of patients with an HIV RNA test result greater than the lower limit of detection (200 copies/mL) among those who received 1 or more HIV tests during the past 6 months.

These outcomes reflect the principle that optimal HIV care exists across a continuum, from timely diagnosis and linkage to care, to initiation of ART, and sustained adherence and retention in care over the life course [34]. They were selected because they have been identified as key quality improvement metrics by Vivent Health for all of its clinical sites. Missed visits are a marker of vulnerability for disengagement from care [35] and may predict loss of viral suppression [36] and subsequent mortality [37]. Viral suppression is the key indicator of a successful response to ART in all HIV care settings. Minimizing viral nonsuppression is, therefore, a main goal of national and global HIV elimination strategies [38].

### Intervention Overview

The ART-C intervention consists of a bundle of digital health-supported services and traditional care coordination activities that can be used outside of the research context. When a clinic begins implementation of ART-C, all clients at the clinic immediately have the opportunity to download the Connections app and use its standard features without sharing any individual-level data with researchers. This is referred to as patient-directed engagement with the app. Individuals who use the Connections app at this level can participate in discussion forums and view resource pages alongside clients receiving services at more than 250 recovery- and prevention-oriented organizations across the United States who subscribe to the Connections app. Patients using the Connections app at this level are not considered to be research participants.

In addition to the standard level of app engagement open to all Vivent Health clients, patients with risk factors for poor engagement in care are invited to access more intensive and personalized features of ART-C as part of a research protocol. These risk factors are described in the formal participant eligibility criteria and capture a patient’s HIV visit attendance history, viral load values, and self-report substance use history. Patients meeting eligibility criteria have access to additional app features and the opportunity to interact with specially trained staff who monitor activity within the Connections app and interact with study participants on a study-specific discussion board. This is referred to as the clinic-supported intervention engagement. These 2 levels of intervention engagement allow the most resource-intensive components of the intervention to be targeted toward patients at greatest risk for viral nonsuppression, while offering all patients the opportunity to participate in the online community fostered by the Connections app.

### Patient-Directed Intervention Components

#### Peer Mentors

Peer-based support is an evidence-based strategy for supporting care engagement [39-41]. It also reduces racial and ethnic disparities in health care by addressing stigma and discrimination [42-44]. Upon implementing ART-C, Vivent Health recruited and trained 6 peer mentors living with HIV who demonstrated resilience in meeting HIV treatment goals and overcoming the effects of discrimination, addiction, or other life challenges. Candidates were identified by soliciting nominations from Vivent Health case managers and health care providers who had been oriented to the goals of the intervention. Peer mentors receive a monthly stipend (US $100) to cover phone and data services and as a token of appreciation for the valuable service they provide. Recruitment and training of peer mentors occur before the intervention starts in each clinic, using modified curricula developed for use by the HRSA-funded AIDS Education and Training Centers.

#### The Connections App

Developed and marketed by CHESS Health, the Connections app offers users anytime and anywhere access to a suite of digital health services, including an online community forum used by people engaged in SUD services across the United States. The recovery-oriented community forum is monitored and moderated by a peer engagement team employed by CHESS Health, consisting of trained peer support specialists who can provide feedback and support to people with SUDs. For the ART-C intervention, the research team worked with CHESS Health to build and deploy a parallel HIV care-oriented community forum, which is moderated by Vivent Health peer mentors and allows for online discussion of topics relevant to patients with and without SUDs, including medication adherence, stigma, and other challenges encountered by people living with HIV. Furthermore, the Connections app contains gamification features, where users can earn points for active engagement in the online community and for extended periods of abstinence from drugs or alcohol. The app also supports private discussions among a smaller number of users in group chats, as well as personal messages between users or between users and staff.

### Clinic-Supported Intervention Components

While the Connections app is available to the full clinic population, more intensive support involving digital health–enhanced, person-centered care coordination can only be delivered to a subset of patients due to staffing and other resource limitations. To maximize the potential impact of the ART-C intervention, the research team consulted with Vivent Health leadership to develop the eligibility criteria provided in Textbox 1 for the clinic-supported arm of the intervention.

Our target study population involves people at risk of viral nonsuppression and lapses in HIV care due to current or past substance use and history of disruptions to HIV care engagement or viral suppression, which align with these criteria.

**Inclusion and exclusion criteria.**

**Inclusion criteria**

- Aged 18 years or more
- Can read and write in English
- Receiving medical care for HIV treatment at Vivent Health (confirmed by electronic health record review)
- At least 1 of the following requirements must be met based on self-report: have a self-reported lapse in medication, which is defined as missing 2 or more doses of medication within a 1-month period; have missed at least 1 appointment while receiving medical care at a Vivent Health clinic; have a history of, or current, substance use disorder, or a report that substance use has caused problems for them at some point in their life

**Exclusion criteria**

- Not currently having a smartphone and unwilling to obtain a smartphone
- Appearing to lack the capacity to consent, as evidenced by an inability to read, write, speak, or otherwise provide informed consent due to acute intoxication or chronic conditions

### Research Care Coordinators

Two new staff members with prior employment experience in HIV medical case management were hired by Vivent Health to be fully dedicated to the ART-C intervention. The research care coordinators have a hybrid role on the Vivent Health care team: they function as research specialists, recruiting, screening, and obtaining informed consent for participants in the clinic-supported intervention, and serve as liaisons between patients who engage with app services and other health care professionals on the patients’ care team. Research care coordinators are credentialed as Vivent Health case managers; have access to patients’ medical records; and, with patient consent, can communicate with medical health care providers, case managers, and other staff about or on behalf of patients enrolled in the ART-C intervention.

### Weekly Check-In Surveys and Red Flag Alerts

Patients enrolled in the clinic-supported intervention are sent a weekly check-in survey via the Connections app that assesses common barriers to care, medication adherence, and drug or alcohol use. The content of the weekly survey was developed and pilot-tested in previous research to provide a near real-time indication of patient experiences that may signal an increased risk of lapses in care [29]. While clients are able to reflect on and report notable barriers from the previous week, the results of these surveys will not be automatically shared with their care team. At the end of the survey, clients are required to opt in or out of having their responses forwarded to their care team for triage. Selecting to opt in means that the clinical research coordinator and care team will adhere to a predetermined escalation procedure for assisting the client with their disclosed barriers.

### Participant Recruitment

On a quarterly basis, the Vivent Health data analytics group will send a list of all patients receiving HIV medical care to the ART-C study team. The list will be used to recruit for the clinic-supported intervention of the study. Patients are contacted through mass messaging campaigns using the MyChart feature of the Epic EHR system, a secure text messaging platform (Artera); direct messaging through MyChart; or direct messaging through the preferred contact method listed in their EHR. For patient privacy, patients who receive messages through mass messaging campaigns will receive a link only to the Connections app download page. Patients who receive direct messaging through MyChart or via their preferred contact method will receive a link to the eligibility survey and app download page. To participate in the study, patients must also have a smartphone and be willing to download the Connections app, which will prompt them to complete a brief questionnaire to assess eligibility for the clinic-supported intervention. Eligible patients will receive a follow-up message asking them to schedule a phone or video appointment with a research care coordinator to review the informed consent document and receive a brief orientation to the ART-C intervention and the Connections app. Patients who do not meet the eligibility criteria will receive a follow-up message informing them that they are not eligible to participate in the clinic-supported arm of the study, but they are encouraged to use the Connections app. People who download the app but do not complete the eligibility screener will receive an additional message reminding them about the opportunity to screen for eligibility for the clinic-supported intervention.

For each of the 8 Vivent Health sites, eligibility assessment and screening will continue until the accrual target is reached or until the end of the 1-year implementation period, whichever occurs first. Participants who enroll but later withdraw or are lost to follow-up will not be replaced by newly enrolled participants.

### Data Collection

We will use the RE-AIM model to guide our overall evaluation of implementation. RE-AIM is a comprehensive framework that assesses 5 dimensions: reach, effectiveness, adoption, implementation, and maintenance [45].

The primary “reach” measure will be the percentage of Vivent Health patients with HIV who have downloaded and used ART-C, along with their sex, racial, and ethnic characteristics. The primary “effectiveness” outcomes (missed HIV care visits and viral nonsuppression) will be evaluated from clinical EHR data documenting HIV viral load results and the status (attended and missed) of scheduled HIV care visits. We will also describe Connections app use and collect quarterly surveys from patients in the clinic-supported intervention. “Adoption” at the patient level is based on research-driven metrics of meaningful use for mHealth, defined as a patient accessing any part of ART-C beyond the home page during a given week in the 12-month period after downloading the app [46].

“Implementation” will be evaluated based on fidelity to key components of ART-C at the clinic level, using quantitative and qualitative methods. We will define fidelity in two dimensions: (1) the amount of the intervention received (ie, use of the dashboard) and (2) the quality of intervention delivery (ie, the intervention participant’s reflections on their experience using the dashboard and the effect it had on their approach to care delivery). “Maintenance” will be defined as follow-up on the effectiveness measures (viral load and missing visit rates) in the 6-month postintervention period.

Using qualitative interviews, we aim to gain rich insights into implementation and adoption processes at various sites from a diverse set of stakeholders involved in implementation. Data will be coded, and the analysis will help us refine the Connections app for future dissemination by determining individual and organizational conditions necessary to promote effectiveness. Interviews will be conducted with multilevel stakeholders, including study participants (10 to 20 interviews), clinic staff identified as change leaders (5 to 10 interviews), and the peer mentors (5 interviews).

### Analytic Strategy: Effectiveness and Maintenance

In the stepped-wedge design, each site functions as its own control or usual care group before the implementation of ART-C. During this period, clinics treat patients with HIV using existing, standard treatment protocols and do not yet have access to the Connections app. To characterize intervention effectiveness, we will model site, time, and intervention status as predictors of primary outcomes using a repeated measures model to control for clustering by individuals and sites [47]. An indicator variable for intervention status will be modeled as 0 before intervention and 1 after ART-C is rolled out at each site. Random effects will be used to model the correlation between individuals within the same cluster (ie, site) in the stepped-wedge design. For a design with 8 clusters, 5 time points, and N individuals sampled per cluster per time interval, Y_ijk_ will be the response corresponding to individual k at time j from cluster i (i in 1,..., 7; j in 1,..., 8; k in 1,..., N) and Y_ij_ will be mean for cluster i at time j:

Y_ij_ = µ+ α_i_ + β_j_ + X_ij_θ

In this equation, α_i_ is a random effect for cluster i, β_j_ is a fixed effect corresponding to time interval j, X_ij_ is an indicator of the treatment status in cluster i at time j (1=intervention; 0=control), and θ is the treatment effect. The individual-level responses of no-show rates and viral suppression are binary, so the cluster-level responses Y_ij_ are proportions with variance σ_ij_^2^_e_ = µ_ij_ * (1 – µ_ij_). A variance inflation factor of 1 + (N – 1) ρ is incorporated into the analysis, where ρ is the intraclass correlation coefficient between individuals within the same cluster. Logistic generalized estimating equation (GEE) models will be determined using R statistical software (version 4.3.3; R Foundation for Statistical Computing) with geeglm (family=binomial) [48].

After establishing the base model, we will include potentially confounding and moderating factors by adding covariates, interaction terms, and weighting methods to the models. Potential clinic-level confounders will include the racial and ethnic categories, gender, age, and diagnosis of a SUD.

Power and sample size calculations will be based on the study by Hemming and Taljaard [49]. For a stepped-wedge design with 8 clusters stepped in 1 to 2 clusters at a time over 5 time periods and an assumed intraclass correlation coefficient of ρ=0.10, the design effect relative to an individual-based randomized controlled trial is 3.0. Preliminary data suggest the percentage of enrolled patients with nonsuppressed viral load (HIV RNA ≥200) before intervention to be 26.7%. On the basis of consultation with clinic leadership and pilot data from a single site, we assume a meaningful effect size to be a 10 percentage point reduction in nonsuppressed HIV RNA tests in the study sites after intervention, to 16.7%. The 8 clinical sites average 526 active patients per site per year for an estimated total of 4208 potential patients. We aim to reach approximately 15% of clinic patients using patient engagement strategies geared toward helping those patients most likely to benefit from the clinic-supported intervention. We hypothesize that the implementation strategy can effectively reach 631 (15%) patients and achieve a 68% improvement in viral load suppression among this highly vulnerable population. On the basis of these assumptions, with 3682 active patients, our power to detect a 10 percentage point reduction in nonsuppressed viral loads will be 98.5% (1–β=.985). The study will be sufficiently powered (>80%) to detect up to a 7 percentage point reduction in patients with nonsuppressed viral load.

### Analytic Strategy: Reach, Adoption, and Implementation

We will assess reach by presenting descriptive statistics of the 8 clinics in the trial and comparing demographic characteristics (sex, race, and ethnicity) between patients using the app versus the overall clinic population using chi-square tests. We will assess factors of patient adoption using logistic GEE models (outcome: any meaningful use each week) and Poisson or negative binomial GEE models (outcome: days of meaningful use per week). Predictors of adoption will include patient sociodemographic factors (age, race and ethnicity, gender, sexual orientation, SUD history) and time-varying factors based on measures collected through weekly and semiannual surveys. Adoption will also be examined as a predictor of individual-level effectiveness outcomes for patients who enroll in the app using logistic GEE models (outcomes: viral nonsuppression and missed visit). Models will account for repeated measures using hierarchical clustering for repeated observations from patients within sites.

Interview and focus group data will be recorded and transcribed. Transcripts will be independently coded by 2 members of the research team, who will meet to discuss and resolve any significant coding inconsistencies. We will manage and analyze qualitative data using NVivo (Lumivero). We will use qualitative analysis to assess adoption at the staff level and use this information to understand potential differences in outcomes between sites related to reach, effectiveness, and implementation outcomes.

### Ethical Considerations

This study was approved by the Health Sciences IRB at the UW-Madison (#2023-0938). Participants who agree to enroll in the clinic-supported arm of ART-C will provide written consent to enter the study. Documentation of consent is kept in the Department of Medicine REDCap (Research Electronic Data Capture; Vanderbilt University), which is compliant with the Health Insurance Portability and Accountability Act (HIPAA). To minimize the risks to participants, they will be provided with a paper or electronic copy of the informed consent when they initially screen as eligible to participate in the study. Participants will be informed that their participation is voluntary, that they may withdraw from the study at any time, and that they may skip any survey questions they are uncomfortable answering. We will also inform participants that being involved or discontinuing the study will not affect their access to services at Vivent Health or any treatment they are receiving. Furthermore, we have a certificate of confidentiality to protect the research team from being compelled to disclose identifiable participant information, even by a court subpoena, in any federal, state, or local civil, criminal, administrative, legislative, or other proceedings.

Study investigators developed a written protocol to assure data accuracy and protocol compliance, including data verification and protocol compliance checks. The data manager at the UW research team, in collaboration with the Department of Medicine IT, ensures that all identifiable data are stored on a UW-Madison secure, restricted drive maintained by the Department of Medicine, and that the storage is compliant with university and federal regulations. Any data generated by this study are only accessible to study team members after providing the IRB and HIPAA compliance officer a copy of their certificates of completion of the UW-Madison Human Subjects and HIPAA privacy rule online trainings. A data safety and monitoring board will not be convened for this study, which is considered a minimal risk implementation study. In lieu of a data monitoring committee, the research team will periodically evaluate the data collected for the purpose of monitoring study progress and identifying potential problems. A template will be used to present the most important summary data points (eg, number of patients screened, enrolled, and withdrawn, as well as the proportion of patients completing various study end points on time). These data will be documented and shared with the study team and principal investigators during monthly team meetings. The principal investigators will determine whether any reported problems identified by study staff represent reportable events and will notify the IRB as appropriate.

Study participants will receive US $30 for completing the baseline, 3-month, 6-month, and 9-month surveys, and US $40 for completing the 12-month survey. The maximum value for compensation for 1 participant in this study will be US $160. Participants may choose to receive their incentive either as a digital gift card, delivered by email or text message via the third-party payment platform Tremendous if they consent, or as a check sent to an address of their choosing. Participants will have the option to update their payment preference throughout the study. Compensation will be provided to participants after confirmed completion of the baseline and quarterly surveys.

### Data Quality Steps and Storage

All data collection tools were created and beta tested collaboratively by study team members who have successfully completed Human Subjects Research training and annual HIPAA certifications. Testing was conducted for ease of use, clarity of questions, skip pattern logic, and duration. Instructions for completing the surveys are provided in writing at the start of the survey. Content experts were consulted during the materials drafting stage to ensure that questions are appropriate for the participant population, do not inflict any unnecessary harm or risk to participants, and were written to capture the necessary data elements. Data collection forms, such as the weekly survey and research surveys, will be directly administered to participants and programmed in a manner that participants are asked to respond to all questions to form a complete dataset, but are given an option to indicate “not applicable” or “I prefer not to answer.” Data forms limit the use of free textboxes to ensure data quality. Data will be coded, and the key linking identities to codes will be stored separately and will only be accessible to key personnel, using HIPAA-compliant servers such as REDCap. Final datasets used for analysis will be distributed by the research project manager or program administrator to study team members via a restricted drive, to which only designated research team members have access.

### Protocol Changes and Communication of Findings

The UW-Madison study team consistently communicates with the participating Vivent Health locations to address any modifications to the study. Communication occurs via in-person or virtual meetings, email and phone communications, and Teams (Microsoft Corporation) for any changes related to protected health information. The research director, research supervisor, and research care coordinators at Vivent Health, who are members of the research team and oversee research projects occurring at Vivent Health, also help disseminate key study protocols and information about changes, problems, and closure of the study throughout the duration of the study.

## Results

In the 2 years before starting ART-C implementation (December 2021 to November 2023), 6710 patients received HIV-related services, including a laboratory test or scheduled visit. After excluding patients who never attended a medical visit, died before the time of study approval, or did not speak English, the resulting sample included 6035 (89.94%) patients who received HIV care in 1 of the 8 participating clinics.

Demographic and clinical characteristics of patients in the baseline sample are provided in Table 1. The majority (75%-88%) of patients at all clinics described their gender as men. The mean age of the patients as of December 2021 ranged from 41.7 (Kansas City) to 48.7 years (Denver). Self-reported race varied geographically. African American and Black individuals comprised the majority of patients in the Milwaukee and St Louis clinics, while most patients in the other 6 clinics reported as White individuals. Hispanic ethnicity was least common in St Louis (60/1409, 4.26%) and most common in Austin (210/754, 27.9%). The prevalence of SUD among active patients ranged from 4.2% (13/307; Kansas City) to 37.4% (82/219; Kenosha).

Table 2 displays the results of the baseline analysis of the 2 main study outcomes, providing the baseline level of HIV care indicators to which future, postintervention results will be compared. Over the 2 years before trial inception, patients had a mean of 4.1 to 7.8 scheduled care visits across sites, and 45% to 70% of them missed 1 or more visits. Patients had an average of 2.2 to 4 HIV RNA tests, and 14% to 37% of them had at least 1 nonsuppressed (>200 copies/mL) HIV RNA test. Multimedia Appendix 1 provides additional patient characteristics, such as HIV transmission route, sexual orientation, employment, and other features of the patient population at the clinic.

**Figure 1:**
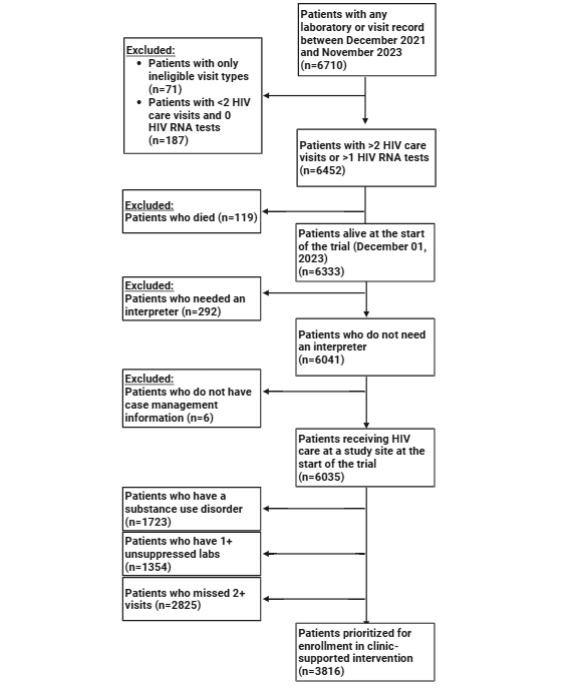
Patients represented in the baseline clinic sample and those prioritized for enrollment in the clinic-supported intervention.

The preliminary estimate for the number of patients who are expected to be eligible for the clinic-supported research protocol is shown in Figure 1. Using the criteria described in the Methods section, we identified 1727 patients with evidence of SUD diagnosis or a previous prescription for a SUD medication during the pretrial period. On the basis of laboratory and visit data in the EHR, 1355 (22.45%) patients had 1 or more nonsuppressed HIV RNA tests, and 2798 (46.36%) patients missed 2 or more HIV care visits. We estimate that the 3816 (63.23%) total patients meeting at least 1 of these 3 criteria will be eligible for the clinic-supported version of the intervention. Table 3 provides the study enrollment and intervention implementation timeline. Primary analyses are expected to be completed by 2028.

**Table 1:** Sociodemographic characteristics of patients receiving HIV care at 8 participating medical home clinics between December 2021 and November 2023 (N=6035).

Characteristics			Medical home clinics														
			Milwaukee (n=1480)		Denver (n=1453)		Green Bay (n=240)		St Louis (n=1409)		Austin (n=754)		Kenosha (n=219)		Madison (n=170)		Kansas City (n=307)
Age (y), mean (SD)			47 (13.4)		48.7 (13.1)		48.1 (12.9)		45.2 (12.3)		42.8 (12.7)		45.7 (14.3)		45.4 (13)		41.7 (12.1)
**Gender, n (%)**																	
	Women	321 (21.69)		121 (8.33)		40 (16.67)		177 (12.56)		93 (12.33)		51 (23.29)		20 (11.76)		60 (19.54)	
	Men	1102 (74.46)		1275 (87.75)		189 (78.75)		835 (59.26)		535 (70.96)		159 (72.6)		139 (81.76)		227 (73.94)	
	Other	44 (3.73)		30 (2.06)		6 (2.5)		31 (2.2)		30 (3.98)		6 (2.74)		7 (4.12)		16 (5.21)	
	Missing	13 (1.1)		27 (1.86)		5 (2.08)		366 (25.98)		96 (12.73)		3 (1.37)		7 (4.12)		4 (1.3)	
**Race, n (%)**																	
	American Indian or Alaska Native	12 (0.81)		49 (3.37)		9 (3.75)		16 (1.14)		8 (1.06)		3 (1.37)		1 (0.59)		5 (1.63)	
	Asian	11 (0.74)		17 (1.17)		3 (1.25)		18 (1.28)		10 (1.33)		0 (0)		5 (2.94)		6 (1.95)	
	Black	798 (53.92)		203 (13.97)		41 (17.08)		741 (52.59)		213 (28.25)		85 (38.81)		42 (24.71)		129 (42.02)	
	Native Hawaiian or Pacific Islander	3 (0.2)		6 (0.41)		1 (0.42)		2 (0.14)		2 (0.27)		1 (0.46)		0 (0)		1 (0.33)	
	White	485 (32.77)		1003 (69.03)		165 (68.75)		572 (40.6)		461 (61.14)		102 (46.58)		108 (63.53)		146 (47.56)	
	Missing	171 (11.55)		175 (12.04)		21 (8.75)		60 (4.26)		60 (7.96)		28 (12.79)		14 (8.24)		20 (6.51)	
**Ethnicity, n (%)**																	
	Hispanic	121 (8.18)		281 (19.33)		44 (18.33)		60 (4.26)		210 (27.85)		31 (14.61)		22 (12.94)		28 (9.12)	
	Non-Hispanic	1279 (86.42)		1100 (75.71)		190 (79.17)		1306 (92.69)		492 (65.25)		177 (80.82)		145 (85.29)		260 (84.69)	
	Missing	80 (5.41)		72 (4.96)		6 (2.5)		43 (3.05)		52 (6.9)		11 (5.02)		3 (1.76)		19 (6.19)	
**Health insurance, n (%)**																	
	Uninsured	48 (3.24)		11 (0.77)		14 (5.83)		18 (1.28)		98 (13)		7 (3.2)		12 (7.06)		15 (4.89)	
	Public Insurance (eg, Medicaid)	263 (17.77)		261 (17.96)		61 (25.42)		246 (17.56)		40 (5.31)		70 (31.96)		33 (19.41)		38 (12.38)	
	Private insurance	197 (13.31)		461 (31.73)		47 (19.58)		309 (21.93)		89 (11.8)		25 (11.42)		40 (23.53)		23 (7.49)	
	Multiple payment types	966 (65.27)		718 (49.42)		117 (48.75)		834 (59.19)		526 (69.76)		117 (53.42)		84 (49.41)		231 (75.24)	
	Missing	6 (0.41)		2 (0.14)		1 (0.42)		2 (0.14)		1 (0.13)		0 (0)		1 (0.59)		0 (0)	
**Substance use disorder history, n (%)**																	
	Yes	409 (27.64)		218 (15)		63 (26.25)		278 (19.73)		108 (14.32)		82 (37.44)		33 (19.41)		13 (4.23)	
	No	1071 (72.36)		1235 (85)		177 (73.75)		1131 (80.27)		646 (85.68)		137 (62.56)		137 (80.59)		294 (95.77)	

**Table 2:** Baseline occurrence of primary outcomes among patients receiving HIV care between December 2021 and November 2023 (N=6035).

Outcomes			Medical home clinics												
			Milwaukee (n=1480)		Denver (n=1453)		Green Bay (n=240)	St. Louis (n=1409)	Austin (n=754)		Kenosha (n=219)		Madison (n=170)		Kansas City (n=307)
**Missed visit outcomes**															
	Clinic visits scheduled, n	11,651		12,134		1305		10,827	6380	1686		993		2236	
	Number of scheduled HIV care visits per patient, mean (SD)	7.1 (5)		7.8 (5.5)		4.1 (2.1)		7.4 (5)	7.6 (5.9)	5.2 (3.3)		4.3 (3.3)		5.7 (3.7)	
	Scheduled visits that were missed (%)	41.13		26.64		21.23		36.63	33.78	38.91		28.60		31.13	
	Patients with 1 or more missed visits (%)	69.57		58.90		45.48		65.85	72.90	58.72		52.16		61.83	
**HIV viral suppression outcomes**															
	HIV RNA tests completed, n	5017		4896		598		5552	2410	691		382		1147	
	Number of completed HIV RNA tests per patient, mean (SD)	3.3 (1.7)		2.7 (1.4)		3.1 (1.9)		2.2 (1.2)	2.1 (1.1)	3.4 (1.8)		4 (2)		3.2 (1.9)	
	HIV RNA tests that were nonsuppressed (%)	9.69		7.31		7.02		11.38	14.11	14.04		10.47		16.30	
	Patients with 1 or more non- suppressed HIV RNA tests (%)	21.65		16.86		13.91		27.05	30.65	26.09		15.73		37.18	

**Table 3:** Study timeline.

	Year 2: March 1, 2023, to February 29, 2024					Year 3: March 1, 2024, to February 28, 2025					Year 4: March 1, 2025, to February 28, 2026					Year 5: March 1, 2026 to February 28, 2027			
	Q^a^1	Q2	Q3	Q4	Q1		Q2	Q3	Q4	Q1		Q2	Q3	Q4	Q1		Q2	Q3	Q4
Kenosha and Madison			EI^b^	EI	EI		EI	I^c^	I	I		I							
Green Bay and Milwaukee					EI		EI	EI	EI	I		I	I	I					
Denver and Kansas City								EI	EI	EI		EI	I	I	I		I		
Austin and St Louis										EI		EI	EI	EI	I		I	I	I

^a^Q: quarter.

^b^EI: enrollment and intervention.

^c^I: intervention.

## Discussion

### Anticipated Findings

People living with SUD are less likely to be retained in HIV treatment for a long term due to various social and structural determinants of health, signaling a need for targeted interventions to support engagement in care. Persistent disparities by race, ethnicity, and socioeconomic status underlie national estimates of viral suppression, fueling the disproportionate burden of new HIV infections occurring in people of color [50,51]. This study aims to characterize the potential impact of digital health combined with peer-based strategies for enhancing engagement in HIV and addiction treatment and care for other complex conditions. We hypothesize that implementation of CHESS within an established network of HIV clinical practices will reduce the occurrence of virologic failure and missed clinic appointments by supporting three needs: (1) facilitating real-time, community-based capture of data reflecting social and behavioral determinants known to precede lapses in HIV care (eg, housing and food insecurity, unhealthy alcohol or drug use, or poor medication adherence); (2) improving engagement in care by increasing social connectedness among patients and between patients and peer mentors; and (3) supporting retention in addiction treatment and mental health care that help maintain engagement in HIV care.

Our analysis of baseline clinical outcomes demonstrated a very high frequency of missed clinic visits across the Vivent Health network. At 7 of the 8 clinics, more than 50% of patients had 1 or more missed visits during the baseline evaluation period from December 2021 to November 2023. Missed visits represent an important target for improving HIV care and HIV-related health disparities, and improving attendance at clinic visits is an important goal of the project. The use of the Connections app may foster beneficial communication and engagement between patients experiencing barriers to care and clinic research care coordinators, who can address obstacles in real time. The findings from this study will contribute a new and innovative set of tools with a high potential impact for improving HIV viral suppression and reducing no-show visits in multiple geographic settings.

### Comparison With Prior Work

Previous studies have demonstrated the feasibility and acceptance of support for medication-taking behavior [52] and increased engagement in HIV care [53], and a growing literature supports the role of mHealth in resource-limited settings [54]. Given that a large proportion of eligible patients are from racial or ethnic minority groups, have public insurance or are uninsured, and have SUDs, an mHealth intervention like the Connections app will likely be both feasible and acceptable among participants. Using a hybrid implementation-effectiveness study design will allow us to characterize the best strategies for implementing the Connections digital health app in a multisite HIV medical home while collecting data needed to assess its impact on important clinical outcomes. By facilitating social support and enhancing motivation, mHealth (digital health) interventions have shown promise for improving outcomes in addiction treatment. This study will build on previous research to determine whether similar benefits can be realized in the field of HIV care. If the intervention proves beneficial, patients may experience improved daily medication adherence and higher levels of sustained viral suppression.

### Strengths and Limitations

A limitation of this study is that the Connections app was derived from an mHealth tool that was originally designed to support care for substance use, not HIV. Given the fact that not all participants may have a SUD, we have added additional components of the Connections app specifically related to HIV to engage such participants. While the centerpiece of the clinic intervention is an established, theory-based mHealth app, the expected impact of the mHealth component derives from its integration with the multiple services and social support delivered as usual care. We aim to maximize opportunities for patient engagement in the mHealth intervention by providing access to the Connections app for all clinic patients living with HIV and implementing a more intensive clinic-supported intervention designed to reach the highest-risk patients. The Connections app will generate alerts based on responses to weekly surveys that indicate a patient is at risk for lapses in care. On the basis of these alerts, research care coordinators will facilitate communication about emerging challenges with members of Vivent Health staff in the participant’s respective clinic.

### Future Directions

Reducing the risk of community-level transmission of HIV requires systems to prevent lapses in ART adherence and improve access to services when patients are not virally suppressed. Moreover, clinic-level interventions must be sustainable over the long term to support patients’ need for lifelong treatment. If this trial demonstrates a beneficial impact on HIV care outcomes as anticipated, future research will be needed to optimize patients’ long-term retention in similar mHealth-based initiatives. Furthermore, cost-benefit studies will be needed to clarify the resources required to sustain novel strategies for supporting vulnerable patients.

## Appendix

Multimedia Appendix 1 Additional sociodemographic and behavioral characteristics of patients at each study clinic during the 2 years before trial implementation, December 2021 to November 2023.

Multimedia Appendix 2 SPIRIT Checklist.

Multimedia Appendix 3 Peer-review report .

## Abbreviations

ART: antiretroviral therapy
ART-C: antiretroviral therapy care coordination
CHESS: Comprehensive Health Enhancement Support System
EHR: electronic health record
GEE: generalized estimating equation
HIPAA: Health Insurance Portability and Accountability Act
HRSA: Health Resources and Services Administration
IRB: institutional review board
mHealth: mobile health
RE-AIM: reach, effectiveness, adoption, implementation, and maintenance
REDCap: Research Electronic Data Capture
RWHAP: Ryan White HIV and AIDS Program
SUD: substance use disorder
UW: University of Wisconsin

## Data Availability

The datasets generated for this analysis and subsequent study are not publicly available due to the presence of protected health information. Data are available upon request and must be submitted on an individual basis to the University of Wisconsin–Madison.
