# Supplementary material for: Health System–Level Implementation of Digital Health Support for People Living With HIV and Substance Use Disorders: Protocol for a Cluster-Randomized, Stepped-Wedge Clinical Trial
Source: JMIR Res Protoc. 2025 Aug 29;14:e69842. doi: 10.2196/69842 (PMC12432469; doi:10.2196/69842)
Supplement: Multimedia Appendix 1 [file resprot_v14i1e69842_app1.docx]

Supplementary Table 1. Additional Sociodemographic and Behavioral Characteristics of Patients in each Study Clinic in the Two Years Prior to Trial Implementation, December 2021 - November 2023

^a^Note: Percentages calculated excluding missing information

|  | Milwaukee N=1480  N (%)^a^ | Denver  N=1453  N (%)^a^ | Green Bay N=240  N (%)^a^ | St. Louis N=1409  N (%)^a^ | Austin N=754  N (%)^a^ | Kenosha N=219  N (%)^a^ | Madison N=170  N (%)^a^ | Kansas City N=307  N (%)^a^ |
| --- | --- | --- | --- | --- | --- | --- | --- | --- |
| **Highest Education Attained** |  |  |  |  |  |  |  |  |
| Less than high school | 199 (26) | 11 (10) | 17 (11) | 1 (20) | 47 (13) | 25 (28) | 5 (6) | 1 (33) |
| High school graduate or GED | 289 (38) | 29 (26) | 66 (44) | 3 (60) | 98 (28) | 40 (44) | 33 (38) | 1 (33) |
| Greater than high school | 273 (36) | 71 (64) | 68 (45) | 1 (20) | 213 (59) | 25 (28) | 49 (56) | 1 (33) |
| **Relationship Status** |  |  |  |  |  |  |  |  |
| Single, divorced, or widowed | 855 (82) | 541 (68) | 128 (76) | 499 (83) | 465 (83) | 88 (72) | 109 (74) | 45 (88) |
| Married or committed relationship | 180 (17) | 235 (30) | 38 (23) | 94 (16) | 89 (16) | 35 (28) | 37 (25) | 6 (12) |
| **Sexual Orientation** |  |  |  |  |  |  |  |  |
| Heterosexual | 566 (43) | 191 (14) | 80 (40) | 278 (32) | 153 (25) | 95 (46) | 29 (23) | 101 (37) |
| Gay, lesbian, or bisexual | 742 (56) | 1130 (83) | 115 (57) | 554 (64) | 433 (71) | 106 (51) | 90 (72) | 164 (59) |
| Other | 17 (1) | 33 (2) | 5 (2) | 32 (4) | 24 (4) | 5 (2) | 6 (5) | 11 (4) |
| **Employment Status** |  |  |  |  |  |  |  |  |
| Unemployed, retired, or disabled | 718 (60) | 336 (50) | 103 (47) | 14 (82) | 344 (52) | 97 (60) | 66 (43) | 2 (50) |
| Full- or part-time work or student | 418 (35) | 317 (47) | 110 (50) | 2 (12) | 304 (46) | 56 (35) | 86 (57) | 2 (50) |
| **Health Status** |  |  |  |  |  |  |  |  |
| Average Charlson Comorbidity Index (CCI) score^b^ | 6.4 (2.3) | 6.1 (2.1) | 6.1 (2.3) | 4.5 (3.3) | 5.5 (2.5) | 6.5 (2.3) | 5.2 (2.8) | 5.2 (2.3) |
| Filled an ART prescription to treat HIV | 1379 (93) | 1381 (95) | 236 (98) | 1307 (93) | 736 (98) | 205 (94) | 158 (93) | 300 (98) |
| **Housing Status** |  |  |  |  |  |  |  |  |
| Stable living arrangements | 1288 (94) | 1180 (92) | 210 (92) | 1168 (99) | 537 (81) | 186 (95) | 142 (92) | 110 (100) |
| Unstable living arrangements | 84 (6) | 102 (8) | 19 (8) | 3 (<1) | 128 (19) | 10 (5) | 12 (8) | 0 (0) |
| **Living in a rural area based on zip code**^c^ | 47 (3) | 39 (3) | 47 (20) | 344 (3) | 22 (3) | 12 (5) | 32 (19) | 11 (4) |
| **HIV Transmission Route** |  |  |  |  |  |  |  |  |
| Male-Male sexual contact | 688 (46) | 611 (42) | 139 (58) | 103 (7) | 409 (54) | 89 (41) | 106 (62) | 74 (24) |
| Injection drug use | 46 (3) | 34 (2) | 6 (2) | 6 (<1) | 23 (3) | 17 (8) | 6 (4) | 10 (3) |
| Heterosexual sexual contact | 404 (27) | 96 (7) | 58 (24) | 39 (3) | 109 (14) | 55 (25) | 28 (16) | 46 (15) |
| Other or unknown route | 341 (23) | 712 (39) | 37 (15) | 1261 (89) | 213 (28) | 58 (26) | 30 (18) | 177 (58) |
| **Substance Use and Mental Health Disorders** |  |  |  |  |  |  |  |  |
| Alcohol use disorder diagnosis or medication prescription | 217 (15) | 93 (6) | 32 (11) | 117 (8) | 74 (10) | 21 (10) | 19 (11) | 20 (6) |
| Opioid use disorder diagnosis or medication prescription | 93 (6) | 74 (5) | 13 (5) | 53 (4) | 52 (7) | 13 (6) | 7 (4) | 9 (3) |
| Substance use disorder diagnosis or medication prescription | 504 (34) | 299 (21) | 71 (30) | 390 (28) | 237 (31) | 83 (38) | 50 (29) | 64 (18) |
| Mental health disorder diagnosis | 911 (62) | 717 (49) | 147 (49) | 827 (59) | 439 (58) | 134 (61) | 123 (72) | 102 (33) |

^b^Minimum CCI index score = 0 and maximum CCI index score = 17

^c^Rurality was determined using the 2010 Rural-Urnab Commuting Area Codes
