# Supplementary material for: Health System–Level Implementation of Digital Health Support for People Living With HIV and Substance Use Disorders: Protocol for a Cluster-Randomized, Stepped-Wedge Clinical Trial
Source: JMIR Res Protoc. 2025 Aug 29;14:e69842. doi: 10.2196/69842 (PMC12432469; doi:10.2196/69842)
Supplement: Multimedia Appendix 3 [file resprot_v14i1e69842_app3.pdf]

SUMMARY STATEMENT
(Privileged Communication)

PROGRAM CONTACT:
MARCY Fitz-Randolph
301-443-9800
marcy.fitz-randolph@nih.gov

Release Date: 12/03/2021
Revised Date:

Application Number: 1 R01 DA055527-01A1
Formerly: 1R01DA055527-01A1

Principal Investigators (Listed Alphabetically):

QUANBECK, ANDREW
WESTERGAARD, RYAN PATRICK (Contact)

Applicant Organization: UNIVERSITY OF WISCONSIN-MADISON

Review Group: PPAH
Population and Public Health Approaches to HIV/AIDS Study Section
AIDS - EXP. REV.

Meeting Date: 11/15/2021
Council: JAN 2022
Requested Start: 03/01/2022
RFA/PA: PAR20-036
PCC: CM/MEF
Dual PCC: A27E
Dual IC(s): AI, HD, MH

Project Title: Clinic-level implementation of mHealth to improve HIV viral suppression for patients with substance use disorders
SRG Action: Impact Score:30 Percentile:21
Next Steps: Visit https://grants.nih.gov/grants/next_steps.htm
Human Subjects: 30-Human subjects involved - Certified, no SRG concerns
Animal Subjects: 10-No live vertebrate animals involved for competing appl.
Gender: 1A-Both genders, scientifically acceptable
Minority: 1A-Minorities and non-minorities, scientifically acceptable
Age: 3A-No children included, scientifically acceptable

Project Year	Direct Costs Requested	Estimated Total Cost
1	493,430	701,673
2	492,035	699,689
3	495,992	705,316
4	499,494	710,296
5	497,625	707,638
TOTAL	2,478,576	3,524,613

ADMINISTRATIVE BUDGET NOTE: The budget shown is the requested budget and has not been adjusted to reflect any recommendations made by reviewers. If an award is planned, the costs will be calculated by Institute grants management staff based on the recommendations outlined below in the COMMITTEE BUDGET RECOMMENDATIONS section.

1R01DA055527-01A1 Westergaard, Ryan

RESUME AND SUMMARY OF DISCUSSION: The applicants seek to develop and integrate an mHealth app and a peer mentoring intervention into an already existing care model in four states using a hybrid type 2 effectiveness-implementation approach to address HIV prevention and care among persons with substance use disorders and ethnic/racial minorities who encounter barriers to accessing prevention and care services. They hypothesize that this integrated mHealth-peer mentoring intervention will have a positive impact on medication adherence, viral suppression, and general engagement in care. The mechanism of action in these outcomes relies on greater community/social connectedness that captures the precursors of disengagement in real time (e.g., food insecurity, alcohol abuse, mental health issues) and can permit early intervention. A cost effectiveness analysis of the intervention will also be performed to determine sustainability. The application is based on very strong premise as mHealth and peer mentoring interventions have shown some preliminary success in increased viral suppression and engagement in care among non-suppressed persons with substance abuse. This application is appropriately grounded in situated IMB model that will guide the development of the intervention. The applicants are very well qualified having the training and experience in research and practice on HIV and substance abuse. They have presented an MPI plan that is very well justified. They are collaborating with an excellent team of multidisciplinary co-investigators who have the complementary skills to conduct this research. The research design is strong; the data collection through weekly diaries provides the opportunity to intervene as outcome are affected; this approach is well supported by preliminary data in the applicants' R34 supporting the ability to conduct weekly surveys with good retention. Likewise, the analyses planned are generally robust. This resubmission was very responsive to the prior review and the applicants have addressed the issues raised (e.g., added a Social/Behavioral scientist on the team; subject fatigue) satisfactorily; the application has been greatly improved. There were, however, some remaining concerns including the following: the cost-effectiveness component of the application is not clearly described; moreover, the applicant seems to use sustainment and cost-effectiveness interchangeably – they are not the same. The use of percent VL suppression in patients with at least one clinic visit in the past year could bias the results towards finding no effect on VL, if most patients who participate are those lost to follow-up; the NIATx model does not seem to have been validated among substance users to show that it can improve persistence in smartphone use. Finally, inclusion criteria for substance users continue to be vague. Despite these concerns, the committee feels that this application can move the field forward in addressing VL among non-suppressed substance users and racial/ethnic minority individuals. As such they assessed the application's potential impact as high.

DESCRIPTION (provided by applicant): In response to PAR-20-036: Getting to Zero: Understanding HIV Viral Suppression and Transmission in the United States, we propose a hybrid type 2 effectiveness-implementation study that will evaluate an innovative clinic-level intervention featuring an evidence-based mobile health (mHealth) application (CHESS) and peer- driven social support. Within the infrastructure of a large HIV/AIDS Service Organization offering an integrated, patient-centered model of care in Colorado, Missouri, Texas and Wisconsin, we will implement an mHealth system designed to close information gaps, build intrinsic motivation, and develop behavioral skills needed for sustained adherence to treatment. Peer support will be provided through activities delivered by patients recruited and trained to serve as peer mentors. The mHealth system and peer mentoring will be integrated into the existing care model, known as the HIV Medical Home. We hypothesize that the integrated intervention will increase the proportion of patients with viral suppression and reduce missed clinic appointments by supporting three needs: (1) It will facilitate real-time, community-based capture of data reflecting social and behavioral determinants known to precede lapses in HIV care (e.g., housing and food insecurity, unhealthy alcohol or drug use, or poor medication adherence); (2) It will improve engagement in care by increasing social connectedness among patients, and between

patients and peer mentors; and (3) It will support retention in addiction treatment and mental health care that help maintain engagement in HIV care. Project Year 1 will be devoted to planning and refinement of the intervention in close collaboration with organizational leaders and people living with HIV who are members of the local communities. Beginning in Project Year 2, we will conduct a stepped wedge cluster randomized trial of the intervention in seven clinics. We will leverage data collected through the integrated electronic health record system serving all seven clinics to test intervention effectiveness on viral suppression and retention in care. We also will conduct an implementation cost analysis and cost-effectiveness analysis to inform future sustainment of the intervention model. The study builds on a solid foundation of prior research demonstrating the promise of mHealth and peer strategies for enhancing engagement in HIV and addiction treatment and care for other complex conditions. The findings from this study, if successful, will contribute a new and innovative set of tools with high potential impact for improving HIV viral suppression in multiple geographic settings.

PUBLIC HEALTH RELEVANCE: People living with substance use disorders and people from racial and ethnic minority groups make up a disproportionate share of new HIV infections in the United States, and once diagnosed, they are less likely to achieve and maintain viral suppression. These disparities reflect complex social and structural determinants of health, which can be addressed by existing medical and social services only if they are accessible, culturally appropriate, and delivered to patients when and where they are needed. This project will test the effectiveness of a combination of strategies, including an evidence-based mobile health app and peer mentoring, that can be integrated into an HIV clinical practice to improve viral suppression and retention in care for vulnerable patients.

CRITIQUE 1

Significance: 2
Investigator(s): 1
Innovation: 1
Approach: 4
Environment: 2

Overall Impact: The proposal addresses the question “how can HIV treatment providers improve viral suppression among patients who are marginally engaged in care or out of care entirely.” The investigators propose a type 2 hybrid effectiveness-implementation stepped wedge RCT to investigate an mHealth app developed by the Center for Health Enhancement Systems Studies (CHESS). They situate their application within a large HIV service organization in 7 facilities in several states (CO, MO, TX, WI). The application relies on the IMB framework and the work follows the EPIS model.

Overall, it is a very thoughtful, well-composed proposal. The premise is very strong—viral suppression, a highly meaningful outcome for both treatment and prevention is time-varying with most people experiencing lapses over the course of a lifetime on treatment. However, with real-time information about social determinants of virologic failure, intervention is possible. The app is designed to identify these determinants in real-time and allow for intervention through a range of social supports and other interventions. This premise is compelling. Strengths around the research plan include 1) an appropriate population to study, 2) an app that has been evaluated in other contexts with appropriate proposed work to tailor it to this population, 3) a reasonable design, and 4) meaningful outcomes (especially viral suppression). The investigator team is strong and well-balanced and the environment (Vivent Health, RWHAP providers) is strong. If effective, it could make a very meaningful impact on viral suppression in this region and beyond. The minor hesitations include 1) reach of an app in this population 2) further clarification on the costing component.

1. Significance:

Strengths

- The premise of this proposal is compelling—that virologic failure, a meaningful outcome for treatment and prevention, is predictable and can be intervened upon in real time through mHealth app that connects people with tailored support and services.
- The CHESS app has been tested and found effective in two other clinical settings (not in HIV populations). A version of the app for people with alcohol use disorders substantially reduced alcohol use disorders and has since been used in many different populations.
- Completed an R34 to evaluate mHealth intervention for improving engagement in HIV care for out-of-care substance using patients. Demonstrated feasibility of following a cohort and improved viral suppression in 2/3 of non-suppressed patients.
- The team has formative work on the feasibility of capturing time-varying social and behavioral determinants of health.
- Foundation of the app in the IMB framework is compelling.
- Use of EPIS is appropriate.
- Builds on mHealth best practices.
- The integration of an app into an already-strong clinical system is compelling.

Weaknesses

- Are there combinations of social and behavioral determinants of viral rebound that together are stronger predictors of virologic failure than each of these variables individually?
- The expected reach of the intervention seems moderate.
- It is unclear whether this app will be strictly for this setting or is easy to adapt to other HIV treatment settings.

2. Investigator(s):

Strengths

- The team has had 9 NIH grants in this space since 2008.
- Westergaard, the PI, is an ID physician/epidemiologist with a research program focused on health disparities, with a major focus on HIV for key populations. Was the PI of a relevant R34 and NIDA-funded award on mHealth among PWID. Strong relevant publication record.
- Quanbeck, MPI, has a background in Systems Engineering with two NIH-funded R01s on mHealth in alcohol-using populations.
- Cockerham, MPI, is an ID physician and researcher with a Master’s degree in clinical research and a vice president at VIvent Health; she has limited research experience.
- Mundt is a health economist with relevant experience.
- Gicquelais is an epidemiologist and Assistant professor with a background in polysubstance use. Given the strengths of the other investigators, it is not clear what her role adds.
- Firnhaber is affiliated with VIvent Denver.

- Seal was newly added to the team as a behavioral/qualitative expert to lead community needs assessment, behavioral intervention.

Weaknesses

- None noted by reviewer.

3. Innovation:

Strengths

- An app that is responsive to a range of multi-level modifiable factors in real-time and before virologic failure is highly innovative and critically important.

Weaknesses

- The investigators expect the app will reach about half of the eligible population, which means it may have limited reach.

4. Approach:

Strengths

- Population: highly appropriate to selectively enroll those who have non-suppressed viral load or those with high risk of non-suppression.
- Intervention: Compelling mHealth intervention integrated into Vivent system.
- Control: Appropriate ethical control population with minor questions noted below.
- Meaningful outcome (viral suppression) measured in the full clinic population, not simply among the app users. If I am understanding correctly, the viral non-suppression measurement is in the full population of the clinics (3682) not simply the 15% expected to participate. This is appropriate as this is clinic-level randomization.
- Figure 1—the study design schema is very helpful.

Weaknesses

- Sustainment and cost-effectiveness are not the same construct but are treated as such. In this study, simply examining use of the intervention after the trial might be a better indicator of sustainment.
- One weakness of a stepped-wedge design is the non-exchangeability of both calendar time and person time. It seems all participants are enrolling in year 2 of the grant period. If this is the case, the people who will be receiving the intervention in year 2 will be different from those in year 3 and 4 with respect to the amount of time they have been on treatment. Similarly, different events could bias different facilities differentially over this period. If time is associated with the outcome viral suppression (either positively or negatively) this could bias the trial. The investigators should acknowledge this limitation and think through approach for addressing it.
- For the primary outcome in Aim 1 (viral non-suppression), how are those without any (or with few) viral load measurements treated? It is suggested that the investigators more carefully consider how to treat missing observations—either through a composite outcome or imputation methods.
- For the cost-effectiveness analysis, considering use of the intervention beyond one year would be more informative to policymakers.

- Costing aim is difficult to follow.

5. Environment:

Strengths

- Strong institutional environment at University of Wisconsin.
- Being based at the institution that developed the app is valuable: Center for Health Enhancement Systems Studies (CHESS).
- Partnership with Vivent Health with its HIV Medical Home Model as a means of providing essential person-centered care is a strong foundation.

Weaknesses

- Unclear the extent to which group has partnered with Vivent Health in the past.

Study Timeline:

Strengths

- The timeline is appropriate, feasible and clearly presented. The first year is dedicated to the preparation phase, the next 2.5 years to the stepped-wedge RCT, and the final 1.5 years to maintenance of the intervention. The start-up activities, anticipated rate of enrollment, planned follow-up are feasible and well justified. There is ample time for data collection, analysis, and dissemination.

Weaknesses

- Are all persons completing four years of person time? It was unclear if people will contribute all the way through year 5.

Protections for Human Subjects:

- A waiver of informed consent to access de-identified data. Participants will consent to participate in Vivent Connect ap.

Data and Safety Monitoring Plan (Applicable for Clinical Trials Only):

- A Data Security and Monitoring Plan (DSMP) will be implemented in accordance with the Health Insurance Portability and Accountability Act (HIPAA). The DSMP is clear and appropriate. The study monitoring, reporting of unanticipated adverse events, and DSMB are all adequate.

Inclusion Plans:

- Sex/Gender: Distribution justified scientifically
- Race/Ethnicity: Distribution justified scientifically
- For NIH-Defined Phase III trials, Plans for valid design and analysis: Not Applicable
- Inclusion/Exclusion Based on Age: Distribution justified scientifically
- all are appropriate.

Vertebrate Animals:

Not Applicable (No Vertebrate Animals)

Biohazards:
Not Applicable (No Biohazards)

Resubmission:

- Responsive to the concerns surrounding addition of social/behavioral scientist; strengthened community engagement strategy; subject fatigue. The cost-effectiveness analysis remained difficult to follow. It is unclear precisely what research question(s) they are addressing.

Resource Sharing Plans:
Unacceptable

- The authors do not intend to share study data, due to the sensitive nature, but is it possible to share de-identified data?

Budget and Period of Support:
Recommend as Requested

CRITIQUE 2

Significance: 2
Investigator(s): 1
Innovation: 3
Approach: 3
Environment: 1

Overall Impact: This clearly written resubmission application proposes to evaluate clinic-level implementation of a multi-component mHealth-centered approach to improve HIV viral suppression for patients with substance use disorders. The research is significant – rigorous prior research suggests that such an intervention could have a substantial impact on treatment outcomes and transmission of HIV. There is rigorous prior research using the CHESS mHealth tools, including by this group of investigators. The research team is strong, and includes the expertise required to accomplish all areas of the proposed studies. While the individual tools employed in this multi-component intervention are not highly innovative, their combination is somewhat novel, and results of a study of these combined tools would advance the field. The proposal is grounded in sIMB, makes use of EPIS for adaptation, and RE-AIM for evaluation. The environment at the University of Wisconsin, Madison is outstanding, and its facilities, resources, and administrative capacity will contribute to the successful implementation of the research. Partner institutions including Vivent Health, the University of Colorado, the Medical College of Wisconsin, and Tulane are also excellent. The proposal was responsive to previous critiques. Important changes include the addition of a sociobehavioral scientist to build out the community engagement aspects of the research, additional background data supporting the researchers' ability to keep participants engaged with a similar CHESS-based intervention despite a moderate data entry burden, and changes to the CEA that will make it easier to compare to other interventions. I noted two score-driving weaknesses in the approach. First, neither the power/sample size estimates nor analysis plans appear to address the potential limitations of varying cluster sizes,

which could substantially reduce power, particularly with large differences in cluster size and a small number of clusters. This is partially mitigated by the fact that the sample size appears to 'over-power' for the primary endpoint, yielding 98.5% power to detect a 10% difference in VL suppression in intervention vs. control periods. Second, the primary endpoint is %VL suppression in patients with at least one clinic visit within the past year. Patients lost to follow-up may be more likely to have unsuppressed VL. If the intervention results in more of these patients attending visits, this could bias toward finding no effect on VL, even if the intervention is having an overall positive effect on both visits and viral load. This is mitigated by the fact that missed visits will be evaluated as a secondary outcome, so the authors may be able to disentangle these effects in their results.

1. Significance:

Strengths

- Rigorous prior research suggests a CHESS-based intervention + case management + peer counseling could have a substantial impact on treatment outcomes.

Weaknesses

- None noted by reviewer.

2. Investigator(s):

Strengths

- Team has the needed skills in HIV medicine, epidemiology, clinical trials, mHealth, and community engagement.
- The addition of a sociobehavioral scientist to support CE activities is a strength of the revised application.

Weaknesses

- None noted by reviewer.

3. Innovation:

Strengths

- The individual components of the intervention including CHESS, case management, and peer-counseling are not highly innovative, but their combination is a strength and could advance the field if tested rigorously.

Weaknesses

- None noted by reviewer.

4. Approach:

Strengths

- Use of situated IMB model to guide intervention content.
- Use of EPIS go guide adaptation of the intervention.
- Use of RE-AIM as an evaluative framework.
- Preliminary data show association between mHealth collected 'red flags' and patient outcomes.

- Preliminary data show high retention in intervention with a comparable study (A-CHESS) using a similar suite of tools.
- Use of CHESS, a well established mHealth tool that has been used successfully in multiple studies, including by this group of investigators.
- Community Leadership Team will inform interpretation of formative data and translation to the adapted intervention.
- CEA outcomes calculating ICER/QALY will provide data comparable to other CEA.

Weaknesses

- Small number of clusters with wide variation in cluster sizes is not directly addressed in sample size calculation or analysis plan – mitigated by Power >>90% for primary outcome.
- Primary endpoint is %VL suppression in patients with at least one clinic visit within the past year. Patients lost to follow-up may be more likely to have unsuppressed VL. If the intervention results in more of these patients attending visits, this could bias toward finding no effect on VL, even if the intervention is having an overall positive effect on both visits and viral load.

5. Environment:

Strengths

- The environment at the University of Wisconsin, Madison is outstanding, and its facilities, resources, and administrative capacity will contribute to the successful implementation of the research.
- Partner institutions including Vivent Health, the University of Colorado, the Medical College of Wisconsin, and Tulane are also excellent.

Weaknesses

- None noted by reviewer.

Study Timeline:

Strengths

- Timeline incorporates plans for start-up activities, recruitment, retention, implementation, analysis, and dissemination of data.

Weaknesses

- None noted by reviewers.

Protections for Human Subjects:

Acceptable Risks and/or Adequate Protections

- Minimal risk - primarily confidentiality - adequate protections.

Data and Safety Monitoring Plan (Applicable for Clinical Trials Only):

Acceptable

- Uses U Wisconsin - Madison DMC.

Inclusion Plans:

- Sex/Gender: Distribution justified scientifically
- Race/Ethnicity: Distribution justified scientifically
- For NIH-Defined Phase III trials, Plans for valid design and analysis: Not applicable
- Inclusion/Exclusion Based on Age: Distribution justified scientifically
- Both sexes, includes both majority and minority populations, adults only, scientifically justified.

Vertebrate Animals:
Not Applicable (No Vertebrate Animals)

Biohazards:
Not Applicable (No Biohazards)

Resubmission:

- Responsive to prior critiques.

Resource Sharing Plans:
Unacceptable

- States that data cannot be shared because of sensitive nature. This seems not to comply with NIH policy, and appropriate protections could be put in place to allow data sharing.

Budget and Period of Support:
Recommend as Requested

CRITIQUE 3

Significance: 2
Investigator(s): 1
Innovation: 2
Approach: 4
Environment:

Overall Impact: The goal of this project is to implement and evaluate an evidence-based mobile health (mHealth) system to improve HIV viral suppression within a multi-site, comprehensive HIV care program. The target population are patients impacted by substance use disorders and related social and behavioral vulnerabilities that result in unacceptably high levels of HIV transmission and persistent racial and ethnic disparities. The investigators propose to use the Comprehensive Health Enhancement Support System (CHESS) mobile health application, a suite of secure, internet-based services delivered by smartphone that promote positive behavior change and provide social support to people engaged in care for HIV and addiction. The goals are to: (1) facilitate real-time, community-based capture of data on social determinants that precede lapses in HIV care, such as unhealthy alcohol and drug use, housing and food insecurity, criminal justice involvement, or psychological distress; (2)

improve engagement in care by increasing social connectedness among patients and between patients and peer mentors; and (3) support retention in addiction treatment and mental health care that help maintain engagement in HIV care. The proposed study is highly significant and the revisions in the current application greatly strengthened the proposal. The investigators are outstanding and the study is innovative. Many of the weaknesses cited by previous reviewers in the Approach were addressed; however, there remain some limitations.

1. Significance:

Strengths

- The proposed study on mHealth implementation to improve HIV viral suppression for patients with substance use disorders addresses the critical problem of poor treatment outcomes among people who use drugs by testing a novel intervention and its goals are significant.
- In response to previous review, the investigators strengthened their patient- and community engagement strategy and will convene a 10-person Community Leadership Team comprised of community members representative of individuals in each of the recruitment clinics who will have an input into the final content, format, and linguistics of the app.
- Other revisions to the proposal also greatly strengthen the application.

Weaknesses

- None noted.

2. Investigator(s):

Strengths

- The PI, Dr. Westergaard, is an infectious disease physician and epidemiologist specializing in health services research and socioeconomic health disparities with a focus on prevention and treatment of HIV for vulnerable populations. In addition to his position as faculty at the University of Wisconsin he also serves as the Chief Medical Officer for the Bureau of Communicable Diseases and has led the COVID-19 response and prevention efforts for the state of Wisconsin. He has served as the PI of several NIDA grants and has collaborated with the Vivent Health, the main field site for the proposed project.
- Dr. Quanbeck, a systems engineer, is a MPI in the proposed application. He serves as the PI for integrating mHealth for alcohol use disorders into clinical practice and has a systems engineering perspective applied in substance abuse treatment.
- The investigative team is accomplished and have led the first implementation science study of mHealth for integrating behavioral health into primary care. In addition, the team has a history of collaboration.
- In this revision of the proposal, a social/behavioral scientist was added who is a long-time collaborator with Dr. Westergaard and with the Vivent Health organization. He has expertise in the conduct of social behavioral formative and intervention research within a multicultural community-based participatory framework. His expertise will greatly improve the proposed application.

Weaknesses

- None noted.

3. Innovation:

Strengths

- The proposal's innovation is grounded in the integration of multi-component interventions including mHealth, case managers and peer mentors for higher risk patients.

Weaknesses

- Previous critique regarding an ad hoc choice of multi-component interventions and lack of clarity of these interventions in prior theoretical basis is still a weakness.

4. Approach:

Strengths

- The proposed interventions are grounded in the investigators' previous studies showing that the use of mHealth and peer navigation interventions are feasible and acceptable for out-of-care HIV patients with active substance use disorders who are known to be at high risk for poor outcomes.
- Several relevant preliminary studies were conducted by the investigators to show that missed visits, injection drug use and low ART adherence preceded viral rebound. Moreover, acceptability of the app was high by case managers and some study participants were willing to share their experiences and advice with others, demonstrating their potential to serve as peer mentors.
- Proposed implementation of a stepped-wedge study design in 7 clinics, a cluster-randomized trial is appropriate and advantageous in clinic-based interventions with diverse geographies and patient volumes, from ~100 to >1,000 patients.
- The sample size calculation indicates the study will have enough power to detect small increments of behavioral change.
- Sustainment/maintenance phase is proposed to address cost-effectiveness, use by the clinic leaders and staff support use of the app, and whether a critical mass of patients will maintain the use of the app.

Weaknesses

- Validation of the NIATx Model among substance users does not appear to have been done to show that it can improve persistence of health-related smartphone app use.
- The inclusion criteria for the study continue to be vague as far as drug abuse is concerned. Patients who are at highest risk for virologic failure and patients with substance use disorders will be included. However, it is not clear that there will be enough power to analyze the data from substance users separately from those who may not be using substances but are at risk for virologic failure for other reasons.
- It is also not clear whether there will be enough power to compare the endpoints for women as compared to men.

5. Environment:

Strengths

- University of Wisconsin-Madison, University of Colorado Denver Anschutz campus and Tulane University School of Public Health and Tropical Medicine offer excellent environment.

- The participating clinics are an excellent environment to perform the proposed study.

Weaknesses

- None noted.

Study Timeline:

Strengths

- Appropriate start-up activities.
- The anticipated rate of enrollment is appropriate.
- Planned follow-up assessments are appropriate.
- Projected timeline is feasible and justified.

Weaknesses

- None noted.

Protections for Human Subjects:

Acceptable Risks and/or Adequate Protections

- No concerns.

Data and Safety Monitoring Plan (Applicable for Clinical Trials Only):

Acceptable

- No concerns.

Inclusion Plans:

- Sex/Gender: Distribution justified scientifically
- Race/Ethnicity: Distribution justified scientifically
- For NIH-Defined Phase III trials, Plans for valid design and analysis: Not applicable
- Inclusion/Exclusion Based on Age: Distribution justified scientifically
- No concerns.

Vertebrate Animals:

Not Applicable (No Vertebrate Animals)

Biohazards:

Not Applicable (No Biohazards)

Resubmission:

The application was very responsive to previous reviews. A social/behavioral scientist was added to the team.

Resource Sharing Plans:

Acceptable

- No concerns.

Budget and Period of Support:

Budget Modifications Recommended (in amount/time)

Recommended budget modifications or possible overlap identified:

- The Community Leadership Team comprised of community members representative of individuals in each of the recruitment clinics will be asked to participate in many of the proposed activities; however, there doesn't appear to be any budget allocation for them.

THE FOLLOWING SECTIONS WERE PREPARED BY THE SCIENTIFIC REVIEW OFFICER TO SUMMARIZE THE OUTCOME OF DISCUSSIONS OF THE REVIEW COMMITTEE, OR REVIEWERS' WRITTEN CRITIQUES, ON THE FOLLOWING ISSUES:

PROTECTION OF HUMAN SUBJECTS: ACCEPTABLE

INCLUSION OF WOMEN PLAN: ACCEPTABLE

INCLUSION OF MINORITIES PLAN: ACCEPTABLE

INCLUSION ACROSS THE LIFESPAN: ACCEPTABLE

COMMITTEE BUDGET RECOMMENDATIONS: The budget was recommended as requested.

Footnotes for 1 R01 DA055527-01A1; PI Name: Westergaard, Ryan Patrick

NIH has modified its policy regarding the receipt of resubmissions (amended applications).See Guide Notice NOT-OD-18-197 at https://grants.nih.gov/grants/guide/notice-files/NOT-OD-18-197.html. The impact/priority score is calculated after discussion of an application by averaging the overall scores (1-9) given by all voting reviewers on the committee and multiplying by 10. The criterion scores are submitted prior to the meeting by the individual reviewers assigned to an application, and are not discussed specifically at the review meeting or calculated into the overall impact score. Some applications also receive a percentile ranking. For details on the review process, see http://grants.nih.gov/grants/peer_review_process.htm#scoring.

MEETING ROSTER

Population and Public Health Approaches to HIV/AIDS Study Section
Healthcare Delivery and Methodologies Integrated Review Group
CENTER FOR SCIENTIFIC REVIEW
PPAH
11/15/2021 - 11/16/2021

Notice of NIH Policy to All Applicants: Meeting rosters are provided for information purposes only. Applicant investigators and institutional officials must not communicate directly with study section members about an application before or after the review. Failure to observe this policy will create a serious breach of integrity in the peer review process, and may lead to actions outlined in NOT-OD-14-073 at https://grants.nih.gov/grants/guide/notice-files/NOT-OD-14-073.html, NOT-OD-15-106 at https://grants.nih.gov/grants/guide/notice-files/NOT-OD-15-106.html, and NOT-OD-18-115 at https://grants.nih.gov/grants/guide/notice-files/NOT-OD-18-115.html, including removal of the application from immediate review.

CHAIRPERSON(S)

BAUERMEISTER, JOSE ARTURO, PHD
PROFESSOR
DEPARTMENT OF FAMILY AND COMMUNITY HEALTH
SCHOOL OF NURSING
UNIVERSITY OF PENNSYLVANIA
PHILADELPHIA, PA 19104

MEMBERS

ABUOGI, LISA LYNN, MD
ASSOCIATE PROFESSOR
DEPARTMENT OF PEDIATRICS
SCHOOL OF MEDICINE
UNIVERSITY OF COLORADO, DENVER
AURORA, CO 80045

AMIRKHANIAN, YURI A, PHD
PROFESSOR
DEPARTMENT OF PSYCHIATRY AND BEHAVIORAL MEDICINE
CENTER FOR AIDS INTERVENTION RESEARCH
MEDICAL COLLEGE OF WISCONSIN
MILWAUKEE, WI 53202

AUDET, CAROLYN, PHD *
ASSOCIATE PROFESSOR
DEPARTMENT OF HEALTH POLICY
UNIVERSITY OF VANDERBILT
NASHVILLE, TN 37203

BALAN, IVAN C, PHD *
RESEARCH PROFESSOR
DEPARTMENT OF BEHAVIORAL SCIENCE AND
SOCIAL MEDICINE
COLLEGE OF MEDICINE
FLORIDA STATE UNIVERSITY
TALLAHASSEE, FL 32310

BALASUBRAMANIAN, RAJI, DSC
ASSOCIATE PROFESSOR
DEPARTMENT OF BIOSTATISTICS AND EPIDEMIOLOGY
SCHOOL OF PUBLIC HEALTH AND HEALTH SCIENCES
UNIVERSITY OF MASSACHUSETTS
AMHERST, MA 01003

BARNIGHAUSEN, TILL, MD
PROFESSOR AND DIRECTOR
HEIDELBERG INSTITUTE OF GLOBAL HEALTH
FACULTY OF MEDICINE AND UNIVERSITY HOSPITAL
UNIVERSITY OF HEIDELBERG
HEIDELBERG 69120
GERMANY

BAUM, MARIANNA K, PHD *
PROFESSOR
DEPARTMENT OF DIETETICS AND NUTRITION
ROBERT STEMPEL COLLEGE OF PUBLIC HEALTH
AND SOCIAL WORK
FLORIDA INTERNATIONAL UNIVERSITY
MIAMI, FL 33199

BAUMAN, LAURIE J, PHD
PROFESSOR
DEPARTMENT OF PEDIATRICS
ALBERT EINSTEIN COLLEGE OF MEDICINE
BRONX, NY 10461

BENDAVID, ERAN, MD, MS *
ASSOCIATE PROFESSOR OF MEDICINE
DEPARTMENT OF MEDICINE
STANFORD UNIVERSITY
STANFORD, CA 94305

BIRKETT, MICHELLE, PHD
ASSISTANT PROFESSOR
DEPARTMENT OF MEDICAL SOCIAL SCIENCES
AND PREVENTIVE MEDICINE
FEINBERG SCHOOL OF MEDICINE
NORTHWESTERN UNIVERSITY
CHICAGO, IL 60611

CHRISTOPOULOS, KATERINA A, MD, MPH *
ASSOCIATE PROFESSOR
HIV/AIDS DIVISION
SAN FRANCISCO GENERAL HOSPITAL
UNIVERSITY OF CALIFORNIA, SAN FRANCISCO
SAN FRANCISCO, CA 94110

DES JARLAIS, DON C, PHD *
PROFESSOR
SCHOOL OF GLOBAL PUBLIC HEALTH
NEW YORK UNIVERSITY SCHOOL OF MEDICINE
NEW YORK, NY 10003

FRYE, VICTORIA, DRPH, MPH *
ASSOCIATE MEDICAL PROFESSOR
DEPARTMENT OF COMMUNITY HEALTH
AND SOCIAL MEDICINE
SCHOOL OF MEDICINE
THE CITY UNIVERSITY OF NEW YORK
NEW YORK, NY 10035

FUJIMOTO, KAYO, PHD
DISTINGUISHED PROFESSOR
DEPARTMENT OF HEALTH PROMOTION
AND BEHAVIORAL SCIENCES
SCHOOL OF PUBLIC HEALTH
UNIVERSITY OF TEXAS HEALTH SCIENCE CENTER
HOUSTON, TX 77030

GALARRAGA, OMAR, PHD *
ASSOCIATE PROFESSOR
DEPARTMENT OF HEALTH SERVICES,
POLICY AND PRACTICE
BROWN UNIVERSITY SCHOOL OF PUBLIC HEALTH
PROVIDENCE, RI 02912

GAMAREL, KRISTINE E, PHD *
ASSISTANT PROFESSOR
DEPARTMENT OF HEALTH BEHAVIOR
AND HEALTH EDUCATION
SCHOOL OF PUBLIC HEALTH
UNIVERSITY OF MICHIGAN
ANN ARBOR, MI 48109

GORMAN, DENNIS, PHD *
PROFESSOR
DEPARTMENT OF EPIDEMIOLOGY AND BIOSTATISTICS
TEXAS A&M SCHOOL OF PUBLIC HEALTH
COLLEGE STATION, TX 77843

HUGHES, JAMES P, PHD *
PROFESSOR
DEPARTMENT OF BIOSTATISTICS
UNIVERSITY OF WASHINGTON
SEATTLE, WA 98195

JENNESS, SAMUEL, PHD, MPH *
ASSOCIATE PROFESSOR
DEPARTMENT OF EPIDEMIOLOGY
ROLLINS SCHOOL OF PUBLIC HEALTH
EMORY UNIVERSITY
ATLANTA, GA 30030

JOSEPH DAVEY, DVORA, MPH, PHD *
ADJUNCT ASSISTANT PROFESSOR
DEPARTMENT OF EPIDEMIOLOGY
FIELDING SCHOOL OF PUBLIC HEALTH
UNIVERSITY OF CALIFORNIA LOS ANGELES
LOS ANGELES, CA 90024

KERRIGAN, DEANNA L, MPH, PHD
PROFESSOR AND CHAIR
DEPARTMENT OF PREVENTION AND COMMUNITY HEALTH
MILKEN INSTITUTE SCHOOL OF PUBLIC HEALTH
GEORGE WASHINGTON UNIVERSITY
WASHINGTON, DC 20052

KLINE, DAVID M., PHD *
ASSISTANT PROFESSOR
BIOSTATISTICS AND DATA SCIENCE
WAKE FOREST SCHOOL OF MEDICINE
WINSTON-SALEM, NC 27157

LUSENO, WINFRED K, PHD
SENIOR RESEARCH SCIENTIST
PACIFIC INSTITUTE FOR RESEARCH AND EVALUATION
CHAPEL HILL, NC 27514

MCCLELLAND, RAYMOND SCOTT, MD
PROFESSOR
DEPARTMENTS OF MEDICINE, EPIDEMIOLOGY,
AND GLOBAL HEALTH
SCHOOL OF MEDICINE
UNIVERSITY OF WASHINGTON
SEATTLE, WA 98104

NIJHAWAN, ANK ELISABETH, MD, MPH *
ASSOCIATE PROFESSOR
INTERNAL MEDICINE, DIVISION OF INFECTIOUS DISEASES
UT SOUTHWESTERN MEDICAL CENTER
DALLAS, TX 75390

NOSYK, BOHDAN, PHD *
ASSOCIATE PROFESSOR AND ENDOWED CHAIR
ECONOMICS OF HIV/AIDS
FACULTY OF HEALTH SCIENCES
SIMON FRASER UNIVERSITY AND
BC CENTRE FOR EXCELLENCE IN HIV/AIDS
VANCOUVER, BC V6Z1Y6
CANADA

OSTERMANN, JAN, PHD *
ASSOCIATE PROFESSOR
DEPARTMENT OF HEALTH SERVICES POLICY
AND MANAGEMENT
ARNOLD SCHOOL OF PUBLIC HEALTH
UNIVERSITY OF SOUTH CAROLINA
COLUMBIA, SC 29208

OUTLAW, ANGULIQUE Y, PHD *
ASSOCIATE PROFESSOR
DEPARTMENT OF FAMILY MEDICINE AND
PUBLIC HEALTH SCIENCES
SCHOOL OF MEDICINE
WAYNE STATE UNIVERSITY
DETROIT, MI 48202

OWORA, ARTHUR HAMIE, PHD *
ASSISTANT PROFESSOR
SCHOOL OF PUBLIC HEALTH
INDIANA UNIVERSITY BLOOMINGTON
BLOOMINGTON, IN 47401

PHO, MAI TUYET, MD, MPH
ASSOCIATE PROFESSOR
DEPARTMENT OF MEDICINE
SECTION OF INFECTIOUS DISEASES AND GLOBAL HEALTH
UNIVERSITY OF CHICAGO MEDICAL CENTER
CHICAGO, IL 60637

ROSENBERG, NORA, PHD *
ASSISTANT PROFESSOR
DEPARTMENT OF HEALTH BEHAVIOR
GILLINGS SCHOOL OF GLOBAL HEALTH
UNIVERSITY OF NORTH CAROLINA
CHAPEL HILL, NC 27599

SALOMON, JOSHUA A, PHD *
PROFESSOR OF HEALTH POLICY
DEPARTMENT OF HEALTH POLICY, SCHOOL OF MEDICINE
CENTER FOR HEALTH POLICY, FREEMAN SPOGLI
INSTITUTE FOR INTERNATIONAL STUDIES
STANFORD UNIVERSITY
STANFORD, CA 94305

SUNDARARAJAN, RADHIKA LU, MD, PHD *
ASSISTANT PROFESSOR
EMERGENCY MEDICINE
WEILL CORNELL MEDICINE
NEW YORK, NY 10065

WITTE, SUSAN S, PHD
PROFESSOR
SCHOOL OF SOCIAL WORK
COLUMBIA UNIVERSITY
NEW YORK, NY 10027

YOUNG, APRIL MARIE, MPH, PHD
ASSOCIATE PROFESSOR
DEPARTMENT OF EPIDEMIOLOGY
COLLEGE OF PUBLIC HEALTH
UNIVERSITY OF KENTUCKY
LEXINGTON, KY 40536

SCIENTIFIC REVIEW OFFICER

GUERRIER, JOSE H, PHD
SCIENTIFIC REVIEW OFFICER
CENTER FOR SCIENTIFIC REVIEW
NATIONAL INSTITUTES OF HEALTH
BETHESDA, MD 20892

EXTRAMURAL SUPPORT ASSISTANT

CORONADO, GISSELL DEL CARMEN
LEAD EXTRAMURAL SUPPORT ASSISTANT
CENTER FOR SCIENTIFIC REVIEW
NATIONAL INSTITUTE OF HEALTH
BETHESDA, MD 20892

* Temporary Member. For grant applications, temporary members may participate in the entire meeting or may review only selected applications as needed.

Consultants are required to absent themselves from the room during the review of any application if their presence would constitute or appear to constitute a conflict of interest.
